# Assessment of glucose metabolism and cellular proliferation in multiple myeloma: a first report on combined ^18^F-FDG and ^18^F-FLT PET/CT imaging

**DOI:** 10.1186/s13550-018-0383-7

**Published:** 2018-04-10

**Authors:** C. Sachpekidis, H. Goldschmidt, K. Kopka, A. Kopp-Schneider, A. Dimitrakopoulou-Strauss

**Affiliations:** 1 Clinical Cooperation Unit Nuclear Medicine, German Cancer Research Center (DKFZ), Im Neuenheimer Feld 280, 69210 Heidelberg, Germany; 2 Department of Internal Medicine V, University Hospital Heidelberg, Heidelberg, Germany; 3 National Center for Tumor Diseases (NCT) Heidelberg, Heidelberg, Germany; 4 Division of Radiopharmaceutical Chemistry, German Cancer Research Center (DKFZ), Heidelberg, Germany; 5 German Cancer Consortium (DKTK), Heidelberg, Germany; 6 Department of Biostatistics, German Cancer Research Center, Heidelberg, Germany

**Keywords:** ^18^F-FDG, ^18^F-FLT, PET/CT, Multiple myeloma

## Abstract

**Background:**

Despite the significant upgrading in recent years of the role of ^18^F-FDG PET/CT in multiple myeloma (MM) diagnostics, there is a still unmet need for myeloma-specific radiotracers. 3′-Deoxy-3′-[^18^F]fluorothymidine (^18^F-FLT) is the most studied cellular proliferation PET agent, considered a potentially new myeloma functional imaging tracer. The aim of this pilot study was to evaluate ^18^F-FLT PET/CT in imaging of MM patients, in the context of its combined use with ^18^F-FDG PET/CT.

**Results:**

Eight patients, four suffering from symptomatic MM and four suffering from smoldering MM (SMM), were enrolled in the study. All patients underwent ^18^F-FDG PET/CT and ^18^F-FLT PET/CT imaging by means of static (whole body) and dynamic PET/CT of the lower abdomen and pelvis (dPET/CT) in two consecutive days. The evaluation of PET/CT studies was based on qualitative evaluation, semi-quantitative (SUV) calculation, and quantitative analysis based on two-tissue compartment modeling. ^18^F-FDG PET/CT demonstrated focal, ^18^F-FDG avid, MM-indicative bone marrow lesions in five patients. In contrary, ^18^F-FLT PET/CT showed focal, ^18^F-FLT avid, myeloma-indicative lesions in only two patients. In total, 48 ^18^F-FDG avid, focal, MM-indicative lesions were detected with ^18^F-FDG PET/CT, while 17 ^18^F-FLT avid, focal, MM-indicative lesions were detected with ^18^F-FLT PET/CT. The number of myeloma-indicative lesions was significantly higher for ^18^F-FDG PET/CT than for ^18^F-FLT PET/CT. A common finding was a mismatch of focally increased ^18^F-FDG uptake and reduced ^18^F-FLT uptake (lower than the surrounding bone marrow). Moreover, ^18^F-FLT PET/CT was characterized by high background activity in the bone marrow compartment, further complicating the evaluation of bone marrow lesions. Semi-quantitative evaluation revealed that both SUV_mean_ and SUV_max_ were significantly higher for ^18^F-FLT than for ^18^F-FDG in both MM lesions and reference tissue. SUV values were higher in MM lesions than in reference bone marrow for both tracers.

**Conclusions:**

Despite the limited number of patients analyzed in this pilot study, the first results of the trial indicate that ^18^F-FLT does not seem suitable as a single tracer in MM diagnostics. Further studies with a larger patient population are warranted to generalize the herein presented results.

**Electronic supplementary material:**

The online version of this article (10.1186/s13550-018-0383-7) contains supplementary material, which is available to authorized users.

## Background

There has been an increasing interest over the past few years regarding the role of imaging in multiple myeloma (MM). This interest has been driven by the considerable technological advances, mostly in cross sectional imaging, that have taken place and by the recent introduction of some very effective novel therapeutic agents in the oncologist’s armamentarium, which have led to unparalleled levels of response in the disease. In this context, the role of molecular imaging with PET has gained significant importance in MM.

In the last years, the position of ^18^F-FDG PET/CT has been drastically upgraded in the management of MM. A number of studies have highlighted the diagnostic and prognostic value of the modality as well as its excellent performance in treatment response evaluation [1–6]. Most recently, the International Myeloma Working Group (IMWG) has recommended the use of ^18^F-FDG PET/CT as a diagnostic tool in patients with active MM, smoldering MM (SMM), and solitary plasmacytoma [7].

Although the vast majority of functional PET imaging studies is performed with ^18^F-FDG, this tracer carries some serious limitations in MM imaging: it demonstrates a false negativity incidence of approximately 11% which is associated with low hexokinase-2 expression in some MM patients, and it shows lower sensitivity than MRI in diffuse bone marrow infiltration, leading potentially to patient misclassifications if used as the only functional imaging technology [1, 8]. Moreover, ^18^F-FDG, as a glucose analog, is generally restricted in oncological imaging by both false positive (inflammation, post-surgical areas, recent use of chemotherapy, fractures, etc.) and false negative results (hyperglycemia, recent administration of high-dose steroids, etc.).

According to the previous, there is a—still unmet—need for myeloma-specific radiotracers. 3′-Deoxy-3′-[^18^F]fluorothymidine (^18^F-FLT) is the most studied cellular proliferation PET agent [9]. ^18^F-FLT is taken up by cells, phosphorylated by thymidine kinase 1, which is upregulated by about tenfold during the S-phase of the cell cycle, and remains trapped intracellularly without being incorporated into DNA. Its kinetics can be described, as in the case of ^18^F-FDG, with a three-compartment model [10–12]. A recent systematic review has shown that ^18^F-FLT PET seems to be a good predictor of early response to systemic-, radio-, and concurrent chemoradiotherapy and that the modality may be developed into a tool for guiding individualization of treatment strategies [13]. Moreover, ^18^F-FLT is considered a potentially new myeloma functional imaging tracer [14].

The aim of this pilot study was to evaluate^18^F-FLT PET/CT in imaging of MM patients, in the context of its combined use with ^18^F-FDG PET/CT.

## Methods

### Patients

This pilot study included eight patients (four male, four female). Four patients were suffering from symptomatic MM and four patients from SMM. Their mean age was 65.8 years (range 53–77 years). Three of the four symptomatic MM patients were suffering from primary, previously untreated myeloma, while one symptomatic MM patient had recently undergone high-dose chemotherapy (HDT) followed by autologous stem cell transplantation (ASCT) and demonstrated nearly complete response, according to the clinical gold standard of the IMWG uniform response criteria for multiple myeloma [15]. Table 1 presents the analytical characteristics of the patients investigated. The analysis was conducted in accordance with the declaration of Helsinki with approval of the ethical committee of the University of Heidelberg and the federal office of radiation protection (BfS). The study is part of a project of a special research area (B9, SFB-TRR 79) funded by the German Research Foundation (DFG).

**Table 1:** Characteristics of the patients enrolled in the study

Patient number	Diagnosis	Gender	Age	^18^F-FDG PET uptake pattern	^18^F-FLT PET distribution
1	SMM	F	76	Negative	Physiologic
2	Symptomatic MM	M	68	Focal (*n* = 3 lesions)	Physiologic
3	SMM	F	77	Focal (*n* = 1 lesion)	Physiologic
4	SMM	F	66	Diffuse	Physiologic
5	Symptomatic MM	M	65	Mixed (*n* = 27 lesions)	Pathologic (*n* = 13 lesions)
6	Symptomatic MM	M	60	Mixed (*n* = 8 lesions)	Pathologic (*n* = 6 lesions)
7	SMM	F	61	Focal (*n* = 9 lesions)	Physiologic
8	Symptomatic MM (pre-treated)	M	53	Diffuse	Physiologic

*MM* multiple myeloma, *SMM* smoldering MM, *F* female, *M* male

### Data acquisition

The double-tracer study in each patient was completed in two consecutive days. For reasons of radiation protection, the patients were intravenously administered with a maximum dosage of 250 MBq ^18^F-FDG (mean = 224 MBq, range = 168–254 MBq) on the first day and respectively a maximum dosage of 250 MBq ^18^F-FLT (mean = 236 MBq, range = 221–248 MBq) on the second day. Data acquisition consisted of two parts for each tracer: the dynamic part (dPET/CT studies of the lower abdomen and pelvis) and the static part (whole-body PET/CT). dPET/CT studies (field of view = 43.2 cm) were performed for 60 min using a 24-frame protocol (10 frames of 30 s, 5 frames of 60 s, 5 frames of 120 s, and 4 frames of 600 s) and a multibed protocol. The use of lower lumbar spine and pelvic entry for the dynamic series is justified by the fact that this anatomic area is regularly used for diagnostic bone marrow biopsies. After the end of the dynamic PET acquisition, the patients were asked to urinate and then additional whole-body static images from the skull to the feet were acquired with duration of 2 min per bed position for the emission scans. A dedicated PET/CT system (Biograph mCT, 128 S, Siemens Co., Erlangen, Germany) with an axial field of view of 21.6 cm with TruePoint and TrueV, operated in a three-dimensional mode, was used. A low-dose attenuation CT (120 kV, 30 mA) was used for attenuation correction of the dynamic emission PET data and for image fusion. A second low-dose CT (120 kV, 30 mA) was performed after the end of the dynamic series covering the area from the skull to the feet in order to avoid patient movement after the dynamic series. All PET images were attenuation corrected, and an image matrix of 400 × 400 pixels was used for iterative image reconstruction. Iterative image reconstruction was based on the ordered subset expectation maximization (OSEM) algorithm with six iterations and 12 subsets. The reconstructed images were converted to standardized uptake value (SUV) images based on the formula: SUV = tissue concentration (Bq/g)/[injected dose (Bq) × body weight (g)] [16].

### Data analysis

Data analysis was based on qualitative (visual) analysis of the PET/CT scans, semi-quantitative evaluation based on SUV calculations, and quantitative analysis of the ^18^F-FDG and ^18^F-FLT PET/CT scans.

Qualitative analysis was based on visual assessment of the PET/CT scans. Two nuclear medicine physicians evaluated the scans on transaxial, coronal, and sagittal images independently from each other. In the case of ^18^F-FDG PET/CT, bone marrow and skeletal foci presenting with tracer uptake higher than that of the surrounding background, for which another benign etiology was excluded, were considered indicative for myeloma. Four patterns of ^18^F-FDG distribution were identified on PET/CT scans: (a) negative pattern, without any pathological tracer accumulation indicative for MM involvement; (b) focal pattern, in which bone marrow foci of increased ^18^F-FDG uptake were considered MM lesions; (c) diffuse pattern, with an “intense,” diffuse bone marrow tracer uptake in maximum intensity projection (MIP) images, without any ^18^F-FDG avid focal lesions; and (d) a mixed pattern, with a combination of diffuse bone marrow uptake and focal bone marrow lesions [17]. Regarding ^18^F-FLT PET/CT, focal skeletal sites with a tracer uptake higher than that of the surrounding background were also defined as indicative for myeloma. All PET-positive findings were compared with the underlying low-dose CT findings.

Semi-quantitative evaluation was based on volumes of interest (VOIs) and on subsequent calculation of SUV values. VOIs were drawn with an isocontour mode (pseudo-snake) and were placed over sites indicative of MM involvement, as well as over reference tissue (http://www.pmod.com/files/download/v31/doc/pbas/4729.htm). Bone marrow of the fifth lumbar vertebra and os ilium if without focal tracer enhancement served as reference tissue.

**Fig. 1:**
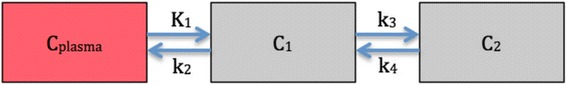
Schematic representation of the three-compartment model applied for ^18^F-FDG and ^18^F-FLT. *K*_1_, *k*_2_, *k*_3_, and *k*_4_ are rate constants (1/min) and describe the directional exchanges between the three compartments (c_plasma_ represents the vascular compartment, c_1_ represents the free and non-specifically bound compartment, and c_2_ represents the trapped compartment). *K*_1_ and *k*_2_ are the inflow and outflow rate constants, respectively between the plasma and the free tracer compartment, and *k*_3_ and *k*_4_ are the rates of phosphorylation and dephosphorylation, respectively

Quantitative evaluation of the dynamic PET/CT data was performed using dedicated software and based on a three-compartment model, which is an accepted approach for assessment of both tracers’ kinetics [11, 18–21]. The three-compartment model leads to the extraction of the kinetic constants: *K*_1_ and *k*_2_, which reflect the transport of the tracers from the plasma to the free and non-specifically bound compartment, and *k*_3_ and *k*_4_, which represent the phosphorylation and dephosphorylation rate of the tracers respectively (Fig. 1). Tracer influx (*K*_i_) is derived from the equation = (*K*_1_ × *k*_3_)/(*k*_2_ + *k*_3_).

The accurate measurement of the input function requires arterial blood sampling. However, the input function for both tracers can be retrieved relatively simplified and noninvasively from the image data with good accuracy according to methods already reported in literature [21–23]. For the input function, the mean value of the VOI data from the common iliac artery, consisting of at least seven sequential ROIs, was used. The recovery coefficient was 0.85 for a diameter of 8 mm. Partial volume correction was performed for small vessels (diameter less than 8 mm) based on phantom measurements of the recovery function using dedicated software [24].

Data were statistically evaluated using the STATA/SE 12.1 (StataCorp) software on an Intel Core (2 · 3.06 GHz, 4-GB RAM) running with Mac OS X 10.8.4 (Apple Inc., Cupertino, CA, USA). The detection rate of lesions by ^18^F-FDG PET and by ^18^F-FLT PET was compared with the Yang test for clustered matched binary data using the R package clust.bin.pair. For comparison of SUV measurements for ^18^F-FDG PET with ^18^F-FLT PET in reference tissue, the reference values from the fifth lumbar vertebra and os ilium for every patient were averaged and compared by paired *t* test. For comparison of SUV_mean_ and SUV_max_ in ^18^F-FLT and ^18^F-FDG, only two patients were available and a patient-wise *t* test was performed. Note that the results cannot be transferred to the patient population. For comparison of SUV_mean_ and SUV_max_ values of both ^18^F-FDG PET and ^18^F-FLT PET between reference and lesions, mixed model analysis was performed with random intercept for the patient using lme(?) from the R package nlme. The results were considered significant for *p* less than 0.05 (*p* < 0.05).

## Results

### Whole-body PET/CT studies

Five patients demonstrated focal, ^18^F-FDG avid, MM-indicative bone marrow lesions, which correlated partially to osteolytic lesions on CT; three of these five patients demonstrated a focal ^18^F-FDG PET pattern, while two of them a mixed pattern. Two patients had a diffuse bone marrow tracer uptake (one of them shortly after HDT), without any ^18^F-FDG avid focal lesions. One patient demonstrated a negative ^18^F-FDG PET pattern (Table 1). In total, 48 ^18^F-FDG avid, focal, MM-indicative lesions were detected with ^18^F-FDG PET/CT. The comparison between ^18^F-FDG PET and the underlying low-dose CT findings in these five patients revealed 18 circumscribed osteolytic lesions in CT that correlated with the ^18^F-FDG avid PET lesions (18/48 lesions, 37.5%). Interestingly, two patients with the initial diagnosis of SMM demonstrated focal bone marrow lesions and were classified as symptomatic MM.

**Fig. 2:**
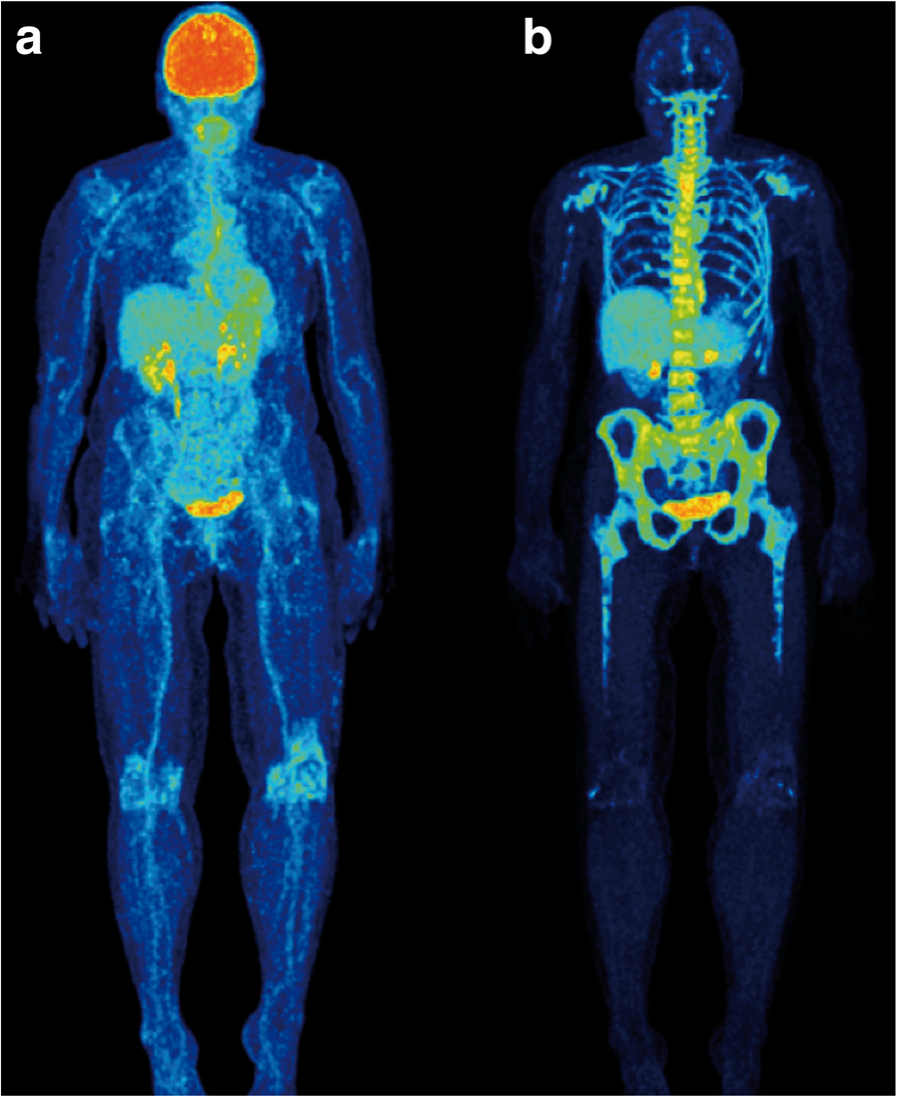
Maximum intensity projection (MIP) ^18^F-FDG PET/CT (**a**) and ^18^F-FLT PET/CT (**b**) of a 76-year-old female SMM patient. No focal, myeloma-indicative lesions detected with either tracer. Normal ^18^F-FLT distribution is characterized by high background activity by the bone marrow compartment (e.g., vertebral column, pelvic skeleton, proximal femora) reflecting the high proliferative activity of hematopoietic cells

**Fig. 3:**
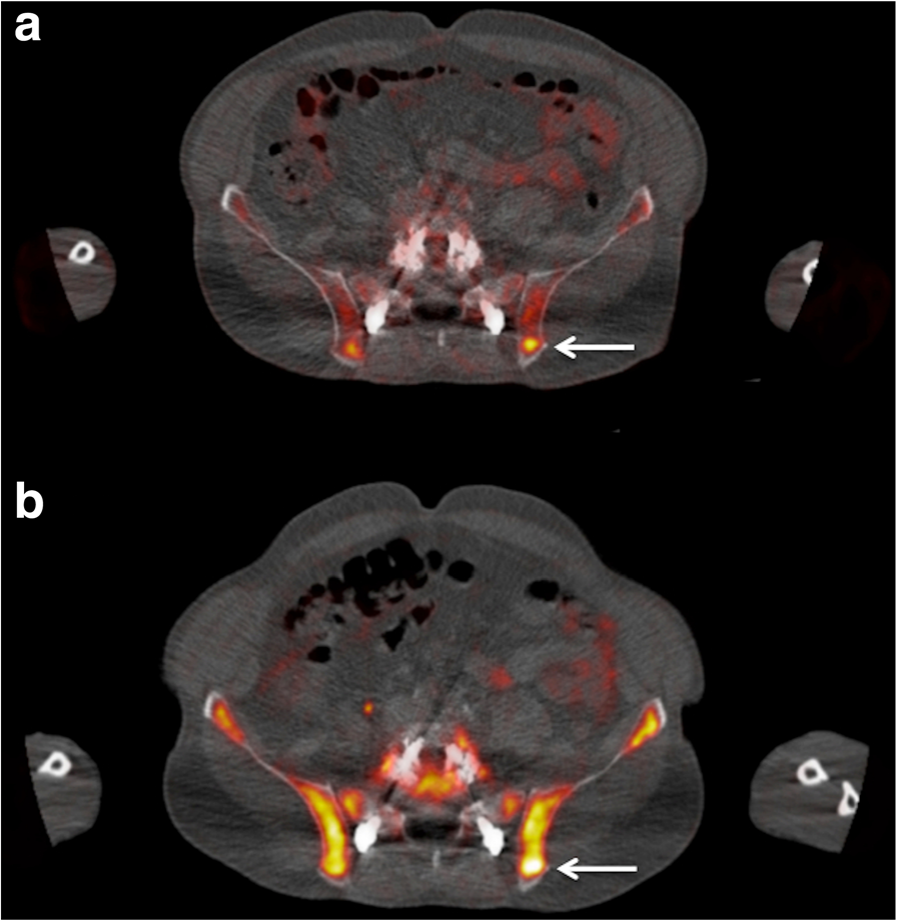
Transaxial ^18^F-FDG PET/CT (**a**) and ^18^F-FLT PET/CT (**b**) images at the pelvic level of a 65-year-old male patient with initial diagnosis of MM. Focally increased ^18^F-FDG accumulation (arrow, **a**) in the left iliac bone, reflecting an active MM lesion (SUV_mean_ = 5.1, SUV_max_ = 7.2). ^18^F-FLT PET/CT also demonstrates focally enhanced tracer accumulation in the same anatomical site (arrow, **b**), higher than the normal bone marrow uptake (SUV_mea*n*_ = 14.1, SUV_max_ = 16.8). Notice the physiologically increased ^18^F-FLT bone marrow uptake rendering evaluation of skeletal lesions challenging (reference SUV_mea*n*_ = 6.5, SUV_max_ = 10.2)

**Fig. 4:**
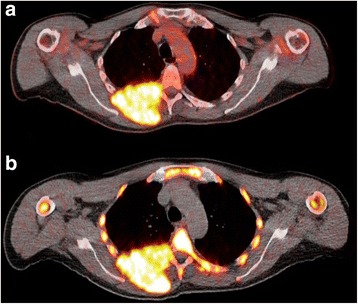
Transaxial ^18^F-FDG PET/CT (**a**) and ^18^F-FLT PET/CT (**b**) images at the thoracic level of a 60-year-old male patient with symptomatic MM. Myeloma lesion of the seventh rib with extramedullary tumor expansion to the surrounding soft tissues and thoracic vertebra after disrupting the cortical bone. The lesion shows increased accumulation in both ^18^F-FDG and ^18^F-FLT PET/CT imaging

**Fig. 5:**
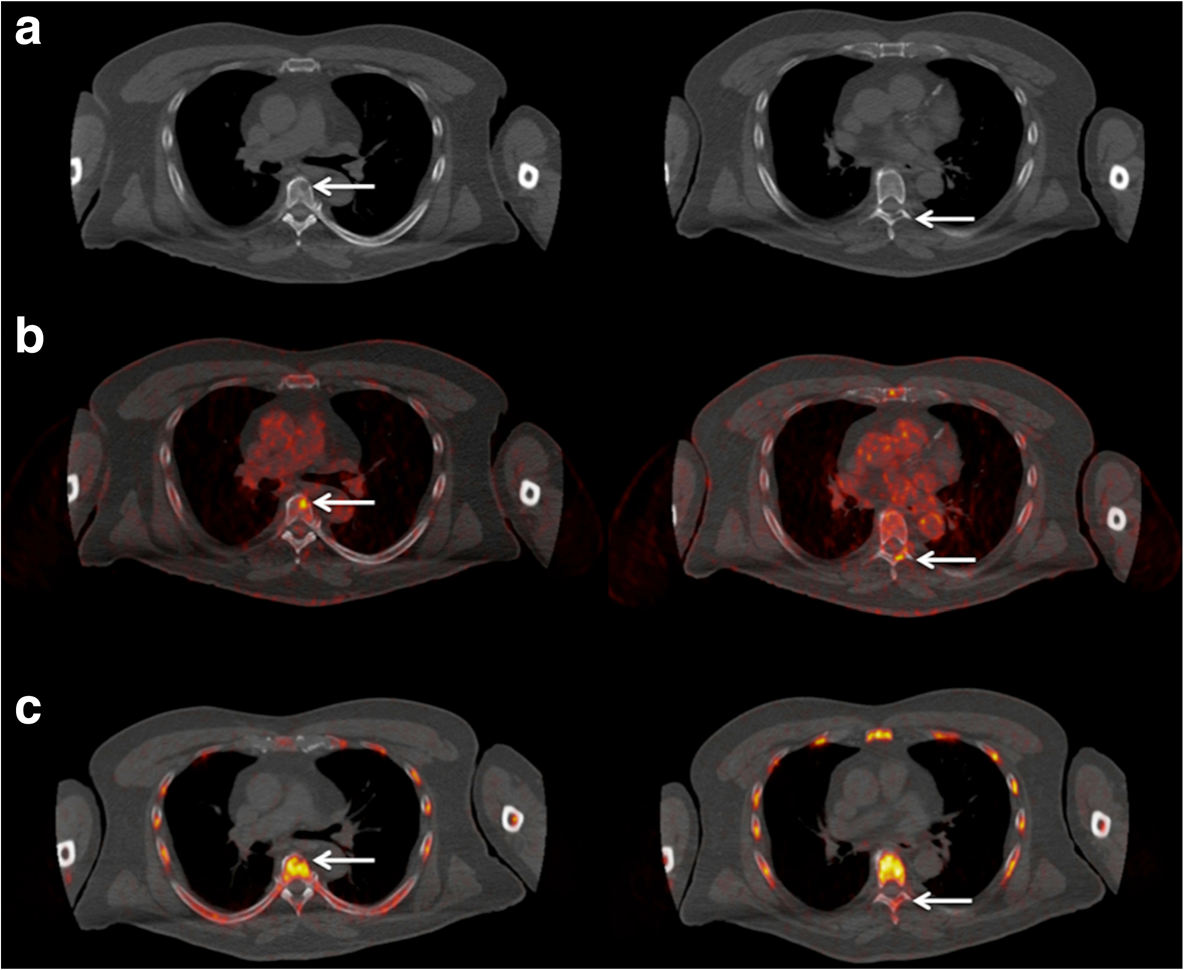
Transaxial low-dose CT (upper row, **a**), ^18^F-FDG PET/CT (middle row, **b**), and ^18^F-FLT PET/CT (lower row, **c**) at the thoracic level of a 65-year-old male patient with initial diagnosis of MM. Intense ^18^F-FDG accumulation (**b**) in osteolytic areas in the sixth thoracic vertebra (left column, **a**) and seventh thoracic vertebra (right column, **a**), reflecting active MM lesions. In contrary, ^18^F-FLT PET/CT revealed reduced tracer accumulation in the MM lesions (**c**), lower than the normal bone marrow uptake

^18^F-FLT PET/CT was characterized by high background activity in the bone marrow compartment (Fig. 2). Two patients demonstrated focal ^18^F-FLT avid lesions with a tracer uptake higher than that of the surrounding bone marrow (Fig. 3). In total, 17 ^18^F-FLT avid, focal, MM-indicative lesions were detected with ^18^F-FLT PET/CT. All 17 ^18^F-FLT avid lesions corresponded to respective ^18^F-FDG avid focal lesions including two lesions with extramedullary tumor expansion to the surrounding soft tissues after disrupting the cortical bone (Fig. 4). Moreover, two ^18^F-FDG-positive lymph nodes demonstrated also increased ^18^F-FLT uptake. Respectively, the comparison between ^18^F-FLT PET and the underlying low-dose CT findings in these two patients revealed 4 osteolytic lesions in CT that correlated with the ^18^F-FLT avid PET lesions (4/17 lesions, 23.5%). Several lesions demonstrated decreased ^18^F-FLT accumulation (lower than the surrounding bone marrow) in anatomical sites that corresponded to focal, ^18^F-FDG avid, osteolytic lesions (Fig. 5). The Yang test showed that the number of ^18^F-FDG avid, focal, MM-indicative lesions was significantly higher (*p* = 0.047) than that of ^18^F-FLT avid lesions. As expected, no pattern of diffuse or mixed tracer uptake (as in the case of ^18^F-FDG PET) could be described on ^18^F-FLT PET, due to the physiologically high ^18^F-FLT accumulation in the bone marrow.

The SUV values of the focal lesions depicted with both tracers were also calculated. For the two patients who had lesions detectable in both modalities, patient-wise *t* test revealed that both SUV_mean_ and SUV_max_ were significantly higher for ^18^F-FLT than for ^18^F-FDG. SUV values were higher in MM lesions than in reference bone marrow for both tracers.

### Dynamic PET/CT studies

**Fig. 6:**
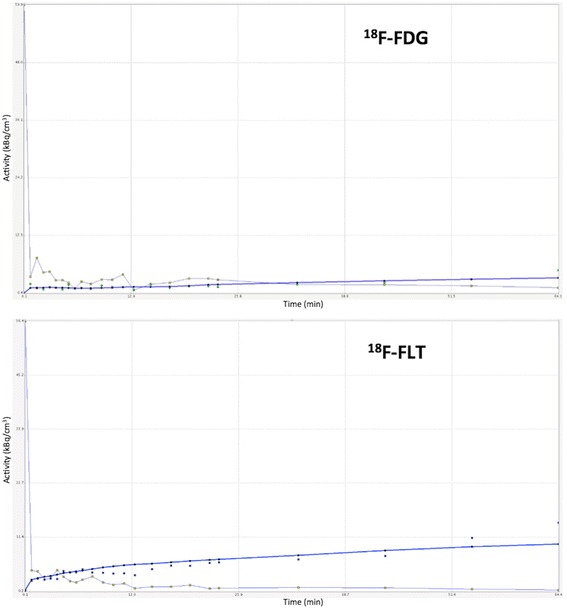
Time activity curves depicting ^18^F-FDG (upper row) and ^18^F-FLT (lower row) concentration during the 60 min of dynamic PET acquisition. The curves are derived from a MM lesion in the os ilium (curve with blue dots) and from the common iliac artery (curve with gold dots) in the respective VOIs. The curves show that both tracers accumulate increasingly in the MM lesions

In total, 12 ^18^F-FDG avid lesions and 5 ^18^F-FLT avid lesions were detected in the lower lumbar spine and pelvic skeleton. Due to the small number of MM-indicative focal lesions, particularly in ^18^F-FLT dPET/CT, no statistical comparison between MM-indicative lesions and reference bone marrow was performed regarding tracers’ kinetics. For the same reason, no comparison between the two tracers’ kinetics was performed. Time activity curves depicting tracers’ concentration in MM lesions during the 60 min of dynamic PET acquisition showed an increasing accumulation in the respective VOIs for both tracers (Fig. 6). The descriptive statistics of the kinetic ^18^F-FDG and ^18^F-FLT data are provided in the supplement (Additional file 1: Tables S1 and Additional file 2: Table S2).

## Discussion

Functional imaging with PET provides the potential of investigating tumor biology at the molecular level after application of several radiotracers. ^18^F-FDG, the workhorse of PET imaging, is a surrogate of glucose utilization. The rationale of ^18^F-FDG application in tumor diagnostics is based on the “Warburg effect,” according to which most cancer cells rely on aerobic glycolysis to generate the energy needed for cellular processes in contrast to normal differentiated cells, which rely primarily on mitochondrial oxidative phosphorylation [25]. Nowadays, ^18^F-FDG PET/CT has become the mainstay for imaging evaluation of several tumor entities. In particular in the case of MM, ^18^F-FDG PET/CT is considered a valuable tool in the work-up of patients with the disease [7, 26]. Nevertheless, as already mentioned, ^18^F-FDG has some well-documented disadvantages that limit its performance in MM evaluation. Thus, the development of myeloma-specific diagnostic imaging agents that could potentially lead to personalized patient management represents a considerable need [27].

Tumor proliferation is a hallmark of the cancer phenotype and one of the useful markers for treatment response evaluation and prognosis in clinical oncology [28]. The radiolabeled thymidine analog ^18^F-FLT can allow noninvasive assessment of tumor proliferation [10]. ^18^F-FLT is incorporated into cells and undergoes phosphorylation by the enzyme thymidine kinase 1, producing ^18^F-FLT monophosphate (^18^F-FLT-MP), which can then be sequentially phosphorylated to form ^18^F-FLT diphosphate (^18^F-FLT-DP) and ^18^F-FLT triphosphate (^18^F-FLT-TP); these phosphorylated products are metabolically trapped inside cells and are not incorporated into DNA. The tracer retention within cells reflects, in part, thymidine kinase activity and is often positively correlated with cellular proliferation [29]. Although its role in everyday clinic has not been yet established, ^18^F-FLT PET has been studied and found to be of clinical significance in several human cancers in diagnosis and treatment response assessment [30–35].

The knowledge regarding application of ^18^F-FLT PET in MM is limited. Up to now, the only existing results have been published by Agool et al., who studied a group of 18 patients with different hematologic disorders, among which, two patients with MM. The authors found that the affected osteolytic areas in these two MM patients demonstrated a low ^18^F-FLT uptake [36]. Despite this lack in literature concerning its use in MM, ^18^F-FLT is considered a promising myeloma functional imaging tracer [14, 37].

The aim of this pilot study was to evaluate ^18^F-FLT PET/CT in imaging of MM patients, in the context of its combined use with ^18^F-FDG PET/CT. Our results show that, if it were used as the only functional imaging modality, ^18^F-FLT PET/CT would have characterized only two patients as demonstrating myeloma-associated, skeletal manifestations. In contrary, ^18^F-FDG PET/CT could reveal skeletal lesions in five of the included patients. Moreover, the number of myeloma-indicative lesions was significantly higher for ^18^F-FDG PET/CT than for ^18^F-FLT PET/CT. An interesting finding of our analysis, which is in line with the results published by Agool et al. [36], is that several affected osteolytic areas demonstrated a tracer mismatch of increased ^18^F-FDG uptake and reduced ^18^F-FLT uptake, indicating a phenomenon of synchronous increased glucose utilization and low proliferation rate in active myeloma lesions (Fig. 5). An explanation for this finding is the fact that MM is in general a tumor with low proliferation rate with a very small fraction of proliferating cells [38]. In agreement with this knowledge, ^18^F-FLT PET/CT showed increased tracer accumulation in a patient with two lesions showing extramedullary expansion to the soft tissue of the chest wall after disrupting the cortical bone (Fig. 4). Given the fact that extramedullary expansion of MM is associated with increased proliferation [39, 40], the demonstration of increased ^18^F-FLT uptake—not only in extramedullary but also in several medullary lesions—indicates an increased proliferation in the myeloma cells of this patient and suggests ^18^F-FLT PET/CT as a potential tool for highlighting the subgroup of MM patients with a hyperproliferative tumor. Unfortunately, no cytogenetic data, potentially demonstrating prognostic unfavorable abnormalities, were available in the particular patient.

Another finding of our analysis is the high background ^18^F-FLT activity in the bone marrow compartment, which further complicates the evaluation of bone marrow lesions in ^18^F-FLT PET/CT. In particular, a diffuse bone marrow infiltration would remain undetected by ^18^F-FLT PET/CT, as observed in our analysis, where two patients showed a diffuse pattern of ^18^F-FDG uptake. Although several causes, such as recent administration of chemotherapy or granulocyte-colony stimulating factor (G-CSF) and anemia, can lead to the diffusely increased bone marrow, ^18^F-FDG uptake is still a finding in PET/CT imaging, which is of particular interest in the case of MM due to the nature of the disease. Nevertheless, its interpretation should be cautious; MRI remains the gold standard for assessment of the degree of bone marrow plasma cell infiltration [41].

Semi-quantitative evaluations showed that tracer uptake, reflected by SUV values, was significantly higher in myeloma-indicative lesions than in reference bone marrow for both ^18^F-FDG and ^18^F-FLT. Moreover, in these cases where lesions were detectable with both tracers (17 lesions), SUV_mean_ and SUV_max_ were significantly higher for ^18^F-FLT than for ^18^F-FDG.

A part of our study focused on the evaluation of the dynamic ^18^F-FDG and ^18^F-FLT PET/CT scans. As already mentioned, a three-compartment model is a reliable approach for characterization of the quantitative behavior of both tracers [11, 18–21]. Unfortunately, due to the small number of MM lesions detected in the pelvic area (particularly for ^18^F-FLT PET/CT), where the dynamic PET acquisition took place, no statistical evaluations regarding tracers’ kinetics were performed.

Limitations exist in this small pilot study. Firstly, the number of patients analyzed does not allow for safe conclusions to be drawn, and further studies with a larger study population are warranted to generalize the herein presented results. Nevertheless, the first results of the trial are not encouraging regarding the application of ^18^F-FLT PET/CT in myeloma diagnostics. Secondly, most of the PET/CT positive findings were not histopathologically confirmed. However, this is usually not possible in the clinical setting. Finally, the dynamic PET/CT studies were confined in the anatomic area of the lower abdomen and pelvis, since whole-body dynamic studies cannot be yet performed. A two-bed position protocol for the dynamic PET acquisition was used, which allows the study of a relatively large field of view of 43.2 cm. Nevertheless, new PET/CT scanners allow dynamic studies over several bed positions by using a continuous bed movement, thus facilitating the use of dynamic protocols and reducing the whole acquisition time.

## Conclusions

This pilot study focusing on the combined use of ^18^F-FLT PET/CT and ^18^F-FDG PET/CT in MM showed that ^18^F-FLT PET/CT failed to demonstrate myeloma-associated skeletal disease in 3/5 patients with bone lesions. Moreover, the number of myeloma-indicative lesions was significantly higher for ^18^F-FDG PET/CT than for ^18^F-FLT PET/CT. Despite the limited number of patients analyzed, the first results of the trial indicate that ^18^F-FLT does not seem suitable as a single tracer in MM diagnostics.

## Additional files

Additional file 1: **Table S1.** Descriptive statistics of kinetic parameters in MM lesions for the tracers ^18^F-FDG and ^18^F-FLT. The parameters K_1_, k_2_, k_3_, k_4_ and influx are expressed in 1/min. (DOCX 55 kb)

Additional file 2: **Table S2.** Descriptive statistics of kinetic parameters in reference bone marrow for the tracers ^18^F-FDG and ^18^F-FLT. The parameters K_1_, k_2_, k_3_, k_4_ and influx are expressed in 1/min. (DOCX 55 kb)
