# Supplementary material for: Assessment of glucose metabolism and cellular proliferation in multiple myeloma: a first report on combined 18F-FDG and 18F-FLT PET/CT imaging
Source: EJNMMI Res. 2018 Apr 10;8:28. doi: 10.1186/s13550-018-0383-7 (PMC5891438; doi:10.1186/s13550-018-0383-7)
Supplement: Supplementary file 1 — Table S1. Descriptive statistics of kinetic parameters in MM lesions for the tracers 18F-FDG and 18F-FLT. The parameters K1, k2, k3, k4 and influx are expressed in 1/min. (DOCX 55 kb) [file 13550_2018_383_MOESM1_ESM.docx]

**Additional file 1: Table S1.** Descriptive statistics of kinetic parameters in MM lesions for the tracers ^18^F-FDG and ^18^F-FLT. The parameters K_1_, k_2_, k_3_, k_4_ and influx are expressed in 1/min.

| Radiopharmaceutical | Parameters | Mean (median) |
| --- | --- | --- |
| **^18^F-FDG** | K_1_ | 0.15 (0.11) |
|  | k_2_ | 0.46 (0.42) |
|  | k_3_ | 0.13 (0.07) |
|  | k_4_ | 0.01 (0.01) |
|  | influx | 0.02 (0.02) |
| **^18^F-FLT** | K_1_ | 0.11 (0.10) |
|  | k_2_ | 0.10 (0.01) |
|  | k_3_ | 0.12 (0.10) |
|  | k_4_ | 0.02 (0.01) |
|  | influx | 0.06 (0.05) |
