# Supplementary material for: Assessment of glucose metabolism and cellular proliferation in multiple myeloma: a first report on combined 18F-FDG and 18F-FLT PET/CT imaging
Source: EJNMMI Res. 2018 Apr 10;8:28. doi: 10.1186/s13550-018-0383-7 (PMC5891438; doi:10.1186/s13550-018-0383-7)
Supplement: Supplementary file 2 — Table S2. Descriptive statistics of kinetic parameters in reference bone marrow for the tracers 18F-FDG and 18F-FLT. The parameters K1, k2, k3, k4 and influx are expressed in 1/min. (DOCX 55 kb) [file 13550_2018_383_MOESM2_ESM.docx]

**Additional file 2: Table S2.** Descriptive statistics of kinetic parameters in reference bone marrow for the tracers ^18^F-FDG and ^18^F-FLT. The parameters K_1_, k_2_, k_3_, k_4_ and influx are expressed in 1/min.

| Radiopharmaceutical | Parameters | Mean (median) |
| --- | --- | --- |
| **^18^F-FDG** | K_1_ | 0.14 (0.07) |
|  | k_2_ | 0.44 (0.34) |
|  | k_3_ | 0.05 (0.05) |
|  | k_4_ | 0.01 (0.01) |
|  | influx | 0.01 (0.01) |
| **^18^F-FLT** | K_1_ | 0.11 (0.10) |
|  | k_2_ | 0.07 (0.02) |
|  | k_3_ | 0.22 (0.13) |
|  | k_4_ | 0.18 (0.16) |
|  | influx | 0.07 (0.06) |
